# Strategies for addressing the needs of children with or at risk of developmental disabilities in early childhood by 2030: a systematic umbrella review

**DOI:** 10.1186/s12916-024-03265-7

**Published:** 2024-02-02

**Authors:** Tracey Smythe, Nathaniel Scherer, Carol Nanyunja, Cally J. Tann, Bolajoko O. Olusanya

**Affiliations:** 1 International Centre for Evidence in Disability, London School of Hygiene & Tropical Medicine, London, UK; 2 Department of Health and Rehabilitation Sciences, Division of Physiotherapy, Stellenbosch University, Cape Town, South Africa; 3 Medical Research Council/Uganda Virus Research Institute and London School of Hygiene and Tropical Medicine Uganda Research Unit, Entebbe, Uganda; 4 Department of Infectious Disease On Epidemiology & International Health, School of Hygiene & Tropical Medicine, London, UK; 5 Neonatal Medicine, University College London NHS Trust, London, UK; 6 Centre for Healthy Start Initiative, Lagos, Nigeria

**Keywords:** Disability, Children under five, Systematic umbrella review, Developmental disabilities

## Abstract

**Background:**

There are over 53million children worldwide under five with developmental disabilities who require effective interventions to support their health and well-being. However, challenges in delivering interventions persist due to various barriers, particularly in low-income and middle-income countries.

**Methods:**

We conducted a global systematic umbrella review to assess the evidence on prevention, early detection and rehabilitation interventions for child functioning outcomes related to developmental disabilities in children under 5 years. We focused on prevalent disabilities worldwide and identified evidence-based interventions. We searched Medline, Embase, PsychINFO, and Cochrane Library for relevant literature from 1st January 2013 to 14th April 2023. A narrative synthesis approach was used to summarise the findings of the included meta-analyses. The results were presented descriptively, including study characteristics, interventions assessed, and outcomes reported. Further, as part of a secondary analysis, we presented the global prevalence of each disability in 2019 from the Global Burden of Disease study, identified the regions with the highest burden and the top ten affected countries. This study is registered with PROSPERO, number CRD42023420099.

**Results:**

We included 18 reviews from 883 citations, which included 1,273,444 children under five with or at risk of developmental disabilities from 251 studies across 30 countries. The conditions with adequate data were cerebral palsy, hearing loss, cognitive impairment, autism spectrum disorder (ASD) and attention-deficit/hyperactivity disorder. ASD was the most prevalent target disability (*n* = 8 reviews, 44%). Most reviews (*n* = 12, 67%) evaluated early interventions to support behavioural functioning and motor impairment. Only 33% (*n* = 10/30) of studies in the reviews were from middle-income countries, with no studies from low-income countries. Regarding quality, half of reviews were scored as high confidence (*n* = 9/18, 50%), seven as moderate (39%) and two (11%) as low.

**Conclusions:**

We identified geographical and disability-related inequities. There is a lack of evidence from outside high-income settings. The study underscores gaps in evidence concerning prevention, identification and intervention, revealing a stark mismatch between the available evidence base and the regions experiencing the highest prevalence rates of developmental disabilities.

**Supplementary Information:**

The online version contains supplementary material available at 10.1186/s12916-024-03265-7.

## Background

There are approximately 53 million children under 5 years of age with developmental disabilities worldwide [1]. Prevalence varies widely across regions and countries, with low- and middle-income countries (LMIC) experiencing a higher prevalence of developmental disabilities than high-income countries [2]. Developmental disabilities are a diverse group of conditions that affect a child’s physical, cognitive and social development [3]. These conditions encompass cerebral palsy, intellectual and learning impairments, epilepsy, hearing and vision impairment and autism spectrum disorder and attention deficit/hyperactivity disorder [4]. Typically, these conditions manifest during early childhood and can have a lifelong impact on children, their families and communities [5]. Children with developmental disabilities may experience delays in reaching developmental milestones, difficulty with social interactions and challenges in accessing and continuing education [6]. These challenges can have long-term consequences, such as decreased employment opportunities and increased dependence on caregivers [7, 8]. Families of children with developmental disabilities may experience financial strain, social isolation and mental health issues [9]. Nevertheless, despite efforts to improve child health and well-being, children with developmental disabilities continue to experience health disparities, social exclusion and limited access to care, particularly in LMIC where the majority of affected children live [10, 11].

In this context, the Sustainable Development Goals (SDGs) aim to achieve universal health coverage, reduce poverty and promote social inclusion, amongst other goals by 2030 [12]. SDG 4 is dedicated to early childhood development and care; specifically, Target 4.2 calls for actions to facilitate school readiness for children with disabilities towards inclusive education. These goals require the identification of children with or at risk of developmental disabilities in the first 5 years of age and the provision of services to address their needs before school entry [13]. However, despite the growing number of children with developmental disabilities, global funding schemes for early childhood development do not adequately address the challenges faced by these children and their families [14].

While services for children with and at risk of developmental disabilities (encompassing prevention, identification and rehabilitation interventions) are often perceived as highly specialised and costly, it is crucial to understand and provide evidence for comprehensive support that may not be so. For instance, evidence-based developmental screening tools integrated into regular early childhood check-ups can streamline identification of potential challenges early on, leveraging existing healthcare infrastructure [15]. This integration eliminates the need for extra appointments, ensuring timely support and contributing to intervention sustainability by utilising the existing network of healthcare professionals, making essential care accessible to a wider population and broadening their impact. Access to care and support should begin with ensuring that routine child health services and education are inclusive of children with disabilities [3]. By embedding inclusivity at this foundational level, we pave the way for a more equitable and supportive environment that can foster better developmental outcomes [16].

Consequently, amidst this drive for equitable access and comprehensive support, there is a growing interest in early identification of developmental disabilities, spurred by a global commitment to equity and inclusive education [17]. However, this poses practical and ethical challenges when suitable services are not available for identified children, particularly in LMIC. The goal of early identification is universal, and some methods and tools used in high-income countries can be beneficial without requiring significant adaptation, depending on the specific disabilities. For example, corrective glasses may not need adaptation to be prescribed in all populations. It is therefore essential to consider contextual differences and carefully assess how evidence-based interventions can be adapted and effectively implemented in various settings to ensure their relevance and effectiveness for the target population. Stigma, discrimination and exclusion further emphasise the need for a transformative approach to early care and support, because they perpetuate societal inequalities, hinder access to essential services and reinforce barriers that impede the holistic development and well-being of children with developmental disabilities [18].

In light of these considerations, this paper sets out to summarise available data on the prevalence of eight prominent developmental disabilities in children younger than 5 years, and the evidence-based interventions for prevention, early detection and rehabilitation. For the purpose of this review, we use the terms “early intervention” and “rehabilitation” for children under 5 with developmental disabilities to refer to timely and targeted strategies that address and mitigate challenges in physical, cognitive, communication and social development. These interventions may encompass a range of services, therapies and support systems designed to enhance their overall well-being, functional abilities and potential for successful integration into society as they grow.

## Methods

This umbrella review was conducted following the Preferred Reporting Items for Overviews of Reviews (PRIOR) statement for conducting umbrella reviews [19]. The protocol for this systematic umbrella review was registered in the International Prospective Register of Systematic Reviews (PROSPERO), reference number CRD42023420099. A comprehensive search of electronic databases was conducted on 14th April 2023, including Embase, Medline, Cochrane Library and PsycINFO, to identify relevant systematic reviews and meta-analyses published in English in the last 20 years (from January 2003 to May 2023). The search strategy included relevant keywords and MeSH terms related to developmental disabilities, prevention, early detection, rehabilitation and children under 5 years of age.

For example: ("PREVENTION" OR "EARLY DIAGNOSIS" OR "EARLY DETECTION" OR "REHABILITATION" OR "EARLY INTERVENTION) AND ("DISABILITY" OR "IMPAIRMENT" OR "DISORDER") AND ("CHILD*" OR CHILD* UNDER FIVE OR CHILD* UNDER 5").

### Inclusion and exclusion criteria

Meta-analyses that met the following criteria were included in this umbrella review:

- Population: Children under 5 years of age diagnosed with or at risk of developmental disabilities, including autism spectrum disorder, attention deficit/hyperactivity disorder, cerebral palsy, epilepsy, hearing loss, intellectual disability, learning disabilities and vision loss. No distinction was made between reviews that evaluated population-based primary studies and those based on a random sample of participants.
- Interventions: Evidence-based interventions for prevention, early detection and rehabilitation of developmental disabilities, including but not limited to medical, behavioural, educational and psychosocial interventions.
- Study design: Systematic reviews and umbrella reviews that included meta-analyses and assessed the effectiveness of interventions for developmental disabilities using rigorous systematic review methodology, including comprehensive literature search, inclusion and exclusion criteria, and quality assessment of included studies.
- Outcome measures: Meta-analyses that report a pooled effect size for child functioning outcomes related to prevention, early detection, or rehabilitation of developmental disabilities, including measures of developmental outcomes, cognitive function, social skills and quality of life.

Systematic reviews that did not meet the above inclusion criteria, such as narrative reviews, opinion pieces, or reviews with low methodological quality, were excluded.

Additional exclusion criteria are meta-analyses that:

(i) Do not include results for children under 5 years of age
(ii) Address secondary health issues in children with disabilities (e.g. oral health for children with cerebral palsy)
(iii) Focus only on parents and do not include outcomes for children with disabilities
(iv) Focus on a specific population group such as children exposed to HIV or malnutrition

We also excluded studies that reported surgical interventions and all invasive medical procedures requiring hospitalisation (such as intrathecal baclofen, scoliosis correction, selective dorsal rhizotomy and umbilical cord blood cell therapy).

### Data extraction

Two independent reviewers screened the titles and abstracts of identified articles for eligibility based on the inclusion and exclusion criteria (TS and either NS or CN). Full-text articles of potentially eligible reviews were retrieved and assessed for inclusion. Disagreements between reviewers were resolved through discussion or consultation with a third reviewer if necessary.

Data from studies retrieved through the systematic search were extracted using Rayaan.ai using pre-defined and piloted forms and exported to Microsoft Excel for analysis. Where studies included data with both child and adult information, only the child information was extracted. Extracted data included the characteristics of included reviews (e.g. authors, publication year, country of origin), population characteristics (e.g. sample size, age range for the meta-analyses undertaken), interventions assessed and outcomes reported. Disaggregated data were managed as follows: where data allowed for disaggregation by children under five, only these specific data were extracted. In cases where data were not disaggregated by age but included children under five, these data were extracted to a separate Excel sheet, and the age range was noted. Extracted data that were not disaggregated were presented as an appendix.

### Quality assessment

The risk of bias (quality) in the included reviews was assessed by the lead author. The Assessment of Multiple Systematic Reviews (AMSTAR2) [20] tool, which is specifically designed for evaluating health intervention research, was utilised to evaluate relevant sources of bias in the reviews. The AMSTAR2 tool takes into consideration the quality of the primary studies included in the meta-analysis, rather than being limited to assessing only the technical aspects of the meta-analysis itself. The AMSTAR2 questionnaire comprises 16 criteria, and reviewers were required to respond with "Yes," "Partial Yes," "No," or "No Meta-analysis" options. The overall quality of the reviews was classified into categories of "critically low," "low," "moderate," or "high."

### Data synthesis

Meta-analyses were grouped by target disability, tabulated and narratively synthesised. Data on effectiveness measures were summarised. Further quantitative meta-analysis was not performed, as studies reported a range of different measures, often in non-representative populations. We present the disaggregated data, with children under 5 years old, with nonaggregate data reported in an appendix.

### Global burden of disease and prevalence of developmental disability

In addition to findings from the included meta-analyses, data were presented on the prevalence of developmental disabilities, as extracted from the most recent prevalence estimates reported by the Global Burden of Disease (GBD) study [21]. This is presently the only source of data on specific developmental disabilities in children under 5 years covering over 200 countries from all world regions [2, 4]. We identified the world regions with the highest prevalence according to the classification of developmental disability and the top ten affected countries. The findings of high-quality reviews were then mapped to the conditions and tabulated.

## Results

**Fig. 1:**
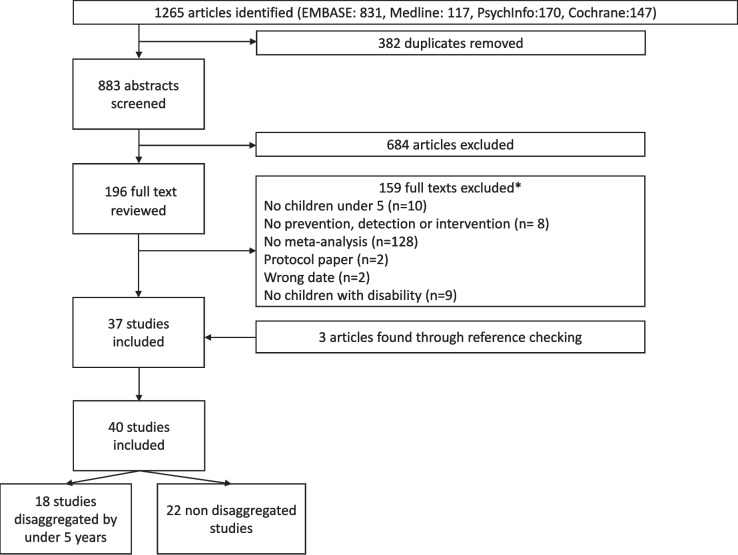
Study selection. *Full texts excluded with reasons provided in Additional File 1

We identified 883 citations in our umbrella review. Of these, 37 met inclusion criteria and three studies were included after manual review (Fig. 1). Amongst the 40 studies, 18 included disaggregated data for children under 5 years, while 22 reviews contained data for children under 5 years, but these were not disaggregated by age.

Eighteen systematic and umbrella reviews explored evidence-based prevention, early detection and early intervention and rehabilitation for 1,273,444 children under five with or at risk of developmental disabilities from 251 studies in 30 countries. Amongst them, half of the reviews (*n* = 9) focused on interventions for children with behavioural disorders, including autism spectrum disorder (ASD) and attention deficit/hyperactivity disorder (ADHD) followed by six reviews (33%) that focussed on children with physical impairment, including cerebral palsy (CP) and neuromotor delay. One review looked at prevention and early intervention, while two focused solely on prevention, and three concentrated on early detection. The remaining 12 reviews (67%) were centred around early intervention (Table 1).

**Table 1:** Summary of characteristics of 18 reviews with data disaggregated for children under five

Category and description		*N* (%)
Disability domain^a^	Motor impairment	66 (26%)
	Cognitive impairment	6 (2%)
	Sensory impairments	19 (7%)
	Behavioural disorders	160 (65%)
Target disability	Developmental delay and at risk	26 (10%)
	Cerebral palsy	46 (18%)
	Hearing impairment	19 (8%)
	ASD	128 (51%)
	ADHD	32 (13%)
Research focus	Prevention	2 (11%)
	Early detection	3 (17%)
	Early intervention	12 (67%)
	Early prevention and intervention	1 (5%)
Country income status^b^	High	20 (67%)
	Middle	10 (33%)
	Low	0 (0%)
Decade of publication	2000	0 (0%)
	2010	9 (50%)
	2020	9 (50%)
Sample size	≤ 100	1 (6%)
	101–1000	5 (28%)
	1001–2000	6 (33%)
	2001–3000	0 (0%)
	> 3000	6 (33%)
Confidence	Low	2 (11%)
	Moderate	7 (39%)
	High	9 (50%)

^a^Number of included studies in meta-analyses, *n* = 251

^b^Reviews included more than one country status

Out of the 30 countries represented in the studies included in the reviews, 20 (67%) were high-income countries, while 10 (33%) were middle-income countries. No low-income countries were represented in the reviews. The highest number of studies came from the USA with a total of 101 studies (40%), followed by the UK with 28 studies (11%) and China and Australia with 24 (10%) and 20 (8%), respectively. Four studies were undertaken in multiple countries. The middle-income countries represented included Bangladesh, China, Egypt, India, Iran, Pakistan, South Africa, Thailand, Tunisia and Turkey. The participant numbers varied across the included reviews, with sample sizes of the meta-analyses ranging from 58 participants with neuromotor delay [22] to 1,023,610 newborns evaluated for early screening for hearing loss [23].

Regarding quality review, this umbrella review includes a majority of reviews (*n* = 16, 89%) with high and moderate confidence (nine reviews and seven reviews respectively) and two reviews (11%) of low confidence (Additional File 2 show the results of the risk of bias assessment of each study with the AMSTAR tool, including the studies that were not disaggregated by age). The most common reasons for low confidence included a combination of the absence of an explicit statement regarding the establishment of review methods before conducting the review, the lack of a list detailing excluded studies and justifying these exclusions, and inadequate investigation of publication bias.

The outcomes and impacts varied across the studies, ranging from reduction in core symptoms for ASD, improved cognitive function and adaptive behaviour, to neuroprotection and improved sitting balance. Table 2 provides a summary of studies focusing on various disabilities and their corresponding evidence for children under 5 years [22–39].

**Table 2:** Study characteristics of published systematic and umbrella reviews exploring prevention, early detection, early intervention and rehabilitation amongst children 5 years of age and younger by target disability

First author (year) [ref]	Target disability	Evidence-based intervention	Intervention details	Country—*n*	Outcomes and impact	Studies in meta-analyses (*n*), Participants (*n*)	Overall confidence
**Motor impairment**							
Inamdar (2021) [24]	Developmental delay and at risk	Early intervention	Physiotherapy plus adjuncts	Egypt—2, South Korea—1, Turkey—1, USA—1	Improvement in sitting with physical therapy plus adjuncts, over physical therapy alone ES = 1.91, (95% CI 0.28–3.54)	5 studies, 146 participants	High
Li (2021) [25]	High risk of brain injury	Early intervention	Early rehabilitation (visual and auditory stimulation, hand- eye coordination training, massage, passive exercise, vestibular exercise training and active guidance activities)	China—13	Early rehabilitation improved development compared to no treatment: OR 4.98 (95% CI 3.66–6.79), improved patient adaptability SMD = 0.63 (95% CI 0.50–0.80) and personal-social scores SMD = 0.79 (95% CI 0.65 to 0.93)	13 studies, 1930 participants	High
Novak (2020) [26]	CP	Prevention and Early Intervention	Interventions for preventing and managing CP in 2019	Finland—1, France—1, Japan—1, New Zealand—1, Netherlands—1, Multiple—2, USA—7,	Antenatal corticosteroids amongst women delivering preterm reduced the rate of CP compared to placebo: RR 0.60 (95%CI 0.34–1.03) Magnesium sulphate amongst preterm neonates reduced the rate of CP compared to placebo: RR 0.68 (95%CI 0.54–0.87) Environmental enrichment improved motor skills compared to standard care: SMD 0.39 (95%CI 0.05–0.72)	14 studies, 7199 participants	High
Shepherd (2018) [27]	CP	Prevention	Therapeutic hypothermia, Prophylactic methylaxanthines (caffeine)	Australia—1, China—1, Germany—1, Multiple—2, New Zealand—1, UK—1, USA—1	Therapeutic hypothermia effective in preventing CP when compared to standard care amongst term neonates with hypoxic-ischaemic neonatal encephalopathy: RR 0.66 (95%CI 0.54–0.82) Prophylactic caffeine effective in preventing CP when compared to standard care: RR 0.54 (95%CI 0.32–0.92)	8 studies, 1525 participants	High
Spittle (2015) [28]	CP	Early intervention	Early developmental intervention post hospital discharge	Australia—2, Canada—1, China—1, Finland—2, Italy—1, Japan—1, Netherlands—1, Norway—3, South Africa—1, Taiwan—1, UK—2, USA—8	Early developmental intervention programmes post hospital discharge improved cognitive outcomes in infancy, developmental quotient: SMD 0.32 (95% CI 0.16–0.47) and at preschool age, intelligence quotient; SMD 0.43 (95% CI 0.32–0.54) compared to standard medical follow-up of preterm infants at infancy	24 studies, 3808 participants	High
Valentin-Gudiol (2013) [22]	Neuromotor delay	Early intervention	Treadmill intervention	Taiwan—1 USA—1	Earlier onset of independent walking (ES) − 1.47 (95%CI − 2.97–0.03)	2 studies, 58 participants	Moderate
**Cognitive Impairment**							
Fischer (2021) [29]	Preterm infant	Prevention	Neuroprotection using erythropoietin (rhEPO)	China—1, Germany—1, Switzerland—1 USA—3	Prophylactic rhEPO for preterm neonates reduced the risk of neurocognitive impairment (defined as MDI < 70 (BSID II) or composite cognitive score < 85 (BSID-III) at 18–26 months’ corrected age from 20 to 14% (OR 0.61, 95%CI 0.39–0.96)	6 studies, 1796 participants	Moderate
**Sensorineural impairment**							
Athe (2022) [30]	Congenital hearing impairment	Early detection	Screening and diagnostic accuracy	Bangladesh—3, India—10, Pakistan—1	Odds of being identified with hearing loss OR:0.52 (95%CI 0.34–0.79)	14 studies, 31,344 participants	Low
Edmond (2022) [23]	Hearing loss	Early detection	Universal newborn hearing screening (UNHS) and diagnostic accuracy	Australia—1, Netherlands—1, UK—1, USA—2,	Improved identification of permanent bilateral hearing loss (PBHL) before 9 months in infants with UNHS programmes compared to infants without UNHS (RR 3.28 (95% CI 1.84–5.85)) Earlier identification of children in the age of identification of PBHL in infants with UNHS compared to infants without UNHS, with mean difference 13.2 months earlier (95% CI − 26.3 to − 0.01)	5 studies, 1,023,610 participants	High
**Behavioural**							
Fuller (2020b) [31]	ASD	Early intervention	Early start Denver model (ESDM) to improve developmental outcomes	Australia—1, Austria—1, China—2, Italy—1, USA—7	Children who received ESDM showed improved outcomes compared to controls: Effect size = 0.36 (*p* = 0.02) (no CI given), driven by improvements in cognition and language	12 studies, 640 participants	Moderate
Hampton (2016) [32]	ASD	Early intervention	Spoken word early intervention	Australia—2, Canada—1, UK—5, USA—18	Early intervention improved spoken language outcomes ES = 0.26 (95%CI 0.11–0.42). Better effect on language outcomes for parent plus clinician delivered interventions (ES = 0.42) compared with parent-only (ES = 0.11) or clinician only (ES = 0.08) delivered interventions	26 studies, 1738 participants	Moderate
Nahmias (2019) [33]	ASD	Early intervention	Community-based early intervention	Australia—7, Canada—1, Israel—2, Italy—2, Norway—1, Sweden—1, Taiwan—1, UK—6, USA—12,	Early intervention improved adaptive behaviour: 0.21 (95%CI 0.13–0.29) and communication outcomes: 0.32 (95% CI 0.24–0.40)	33 studies, 1713 participants	Moderate
Nevill (2016) [34]	ASD	Early intervention	Parent-mediated intervention	Australia—3, Canada—1, Netherlands—1, Thailand—1, UK—3, USA—10,	Parent-mediated interventions improved communication-language 0.16 (95%CI 0.02–0.31) and socialisation 0.22 (95%CI 0.09–0.36)	19 studies, 1025 participants	Low
Reichow (2018) [35]	ASD	Early Intervention	Early intensive behavioural intervention	UK—2, USA—3	Improved adaptive behaviour: MD = 9.58 (95%CI 5.57–13.60), improved IQ: MD = 15.44, (95%CI 9.29–21.59), expressive language: SMD = 0.51 (95%CI 0.12–0.90), receptive language: SMD = 0.55 (95%CI 0.23–0.87)	5 studies, 219 children	High
Sanchez-Garcia (2019) [36]	ASD	Early detection	18 screening tools evaluated	Australia—1, Belgium—1, Japan—3, Norway—1 UK—1, Spain—1, Sweden—1, USA—5	Pooled sensitivity was 0.72 (95% CI 0.61–0.81), and the specificity was 0.98 (95% CI 0.97–0.99) for diagnostic tests for early detection of ASD	14 studies, 191,803 participants	Moderate
Shephard (2022) [37]	ADHD	Early Intervention	Neurocognitive and behavioural intervention	Belgium—1, Canada—2, China—1, Iran—1, Italy—2, New Zealand—3, UK—5, USA—16, Tunisia—1	Intervention-related improvements in ADHD symptoms SMD = 0.43 (95%CI 0.22–0.64) and working memory SMD = 0.37 (95%CI 0.06–0.69)	32 studies, 3848 participants	High
Tachibana (2017) [38]	ASD	Early intervention	Behavioural, social communication and multi-modal interventions	Australia—1, Canada—1, Japan—1, Norway—1, UK—2, USA—2	Improved reciprocity of social interaction towards others’ SMD: 0.53 (95%CI 0.29–0.78)	8 studies, 418 children	High
Wang (2022) [39]	ASD	Early intervention	Early start Denver model	Australia—1, China—5 USA—5	Intervention improved autism symptoms ES: g = 0.27 (95%CI 0.02–0.53) and language ES: 0.28 (95%CI 0.002–0.56)	11 studies, 624 participants	Moderate

*CP* cerebral palsy, *ES* effect size, *OR* odds ratio, *MD* mean difference, *RR* risk ratio, *SMD* standardised mean difference

Data that were not disaggregated are presented in Additional file 3 [40–61].

### Cerebral palsy

Globally, approximately 8 million (95% uncertainty interval [UI] 7,113,334–9,231,577 children younger than 5 years had CP in 2019, with the highest burden being in the African Region (2.7million) and Southeast Asia (2.4million) [21]. Amongst the six (33%) reviews that examined prevention and early intervention for CP, only two [27, 28] included data from a country ranking within the top ten highest prevalence countries, specifically China. Four reviews focussed on early intervention, one on prevention, and one on prevention and early intervention. Amongst preterm infants, antenatal corticosteroids, magnesium sulphate and prophylactic caffeine were all found to significantly reduce the risk of cerebral palsy when compared to placebo or standard care. Likewise, therapeutic hypothermia amongst term neonates with hypoxic-ischemic encephalopathy significantly reduced the risk of motor impairment at 18 months. Improved cognitive outcomes were seen during early childhood (age 2–3 years) following a variety of early developmental interventions, such as early rehabilitation (that included sensory stimulation, co-ordination training) and environmental enrichment. This effect continued to preschool age (4–5 years) (Table 3).

**Table 3:** Burden of developmental disabilities in children under 5 years and summary of evidence by condition

**Condition**	**Estimated Global Burden in 2019 (95% UI)**^**22**^	**Highest burden regions [prevalence]**	**Top ten countries**	**Evidence-based interventions for children with disabilities under 5**		
				**Prevention**	**Early detection**	**Inclusive early intervention and rehabilitation**
Cerebral palsy^b^	8,071,408 (7,113,334–9,231,577)	African Region [2.7 m], Southeast Asia [2.4 m]	India, China, Pakistan, Nigeria, Bangladesh, Ethiopia, DR-Congo, Brazil, Tanzania and Indonesia	**Amongst preterm neonates:** Antenatal corticosteroids: RR 0.60 (95%CI 0.34–1.03) Magnesium sulphate: RR 0.68 (95%CI 0.54–0.87) Prophylactic caffeine: RR 0.54 (95%CI 0.32–0.92) **Amongst term neonates:** Neonatal hypothermia: RR 0.66 (95%CI 0.54–0.82)	-	Environmental enrichment: SMD 0.39 (95%CI 0.05–0.72) Physical therapy plus adjuncts: ES 1.91 (95% CI 0.28–3.54) Early rehab improves development: OR 4.98 (95% CI 3.66–6.79) Earlier onset of independent walking: ES 1.47 (95%CI − 2.97–0.03)
Cognitive impairment^b^	16,057,584 (11,515,194–20,980,652)	Southeast Asia [6.3 m], African Region [3.3 m]	India, China, Pakistan, Nigeria, Ethiopia, Indonesia, DR-Congo, Egypt, Afghanistan and USA	**Amongst preterm neonates:** Prophylactic rhEPO reduced the risk of neurocognitive impairment at 18–26 months’ corrected age: OR 0.61 (95%CI 0.39–0.96)	-	-
Hearing Loss^a^	14,148,322 (12,036,835–16,216,298)	Sub-Saharan Africa [4.4 m], South Asia [3.9 m]	India, China, Nigeria, Pakistan, Bangladesh, DR-Congo, Indonesia, Ethiopia, Brazil and USA	-	Identification of hearing loss with universal newborn hearing screening: 13.2 months earlier (95% CI − 26.3 to − 0.01)	-
Attention-Deficit/Hyperactivity Disorder^a^	1,367,582 (898,677–1,947,054)	East Asia and Pacific [0.5 m], South Asia [0.2 m]	China, India, Nigeria, USA, Ethiopia, DR-Congo, Egypt, Brazil, Indonesia and Iram	-	-	Neurocognitive and behavioural interventions improve ADHD symptoms SMD = 0.43 (95%CI 0.22–0.64) and working memory SMD = 0.37 (95%CI 0.06–0.69)
Autism Spectrum Disorder^a^	2,912,437 (2,418,074–3,461,585)	Sub-Saharan Africa [0.8 m], East Asia and Pacific [0.7 m]	India, China, Nigeria, Pakistan, Indonesia, USA, Ethiopia, Brazil, Bangladesh and DR-Congo		Diagnostic tests: sensitivity: 0.72 (95% CI 0.61–0.81), specificity: 0.98 (95% CI 0.97–0.99)	Spoken word ES = 0.26 (95%CI 0.11–0.42) Community based ES = 0.21 (95%CI 0.13–0.29) Parent-mediated ES = 0.16 (95%CI 0.02–0.31) Intensive behavioural MD = 9.58 (95%CI 5.57–13.60) Behavioural and social communication interventions SMD: 0.53 (95%CI 0.29–0.78) Early Start Denver Model ES = 0.27 (95%CI 0.02–0.53)

Sources of Prevalence Estimates: ^a^Institute for Health Metrics and Evaluation, GBD 2019 (https://vizhub.healthdata.org/gbd-results/) and ^b^WHO Rehabilitation Need Estimator (https://vizhub.healthdata.org/rehabilitation/)

### Cognitive impairment

Approximately 16 million (95% UI 11,515,194–20,980,652) children under 5 years had cognitive impairment worldwide, with the highest burden in Southeast Asia (6.3 million) and the African Region (3.3 million) [21]. China and the USA are the sole nations amongst the top ten with the highest prevalence of cognitive impairment represented in one systematic review that targeted prevention of cognitive impairment. This single systematic review explored prevention of cognitive impairment in preterm neonates and found prophylactic erythropoietin (rhEPO) reduced the risk of neurocognitive impairment at 18–26 months [29]. There were no studies disaggregated for children under five with cognitive impairment regarding early detection or inclusive early intervention and rehabilitation.

### Hearing loss

There were over 14 million (95% UI 12,036,835–16,216,298) children under five with hearing loss, with the highest burden in Sub-Saharan Africa (4.4million) and South Asia (3.9million) [21]. Amongst the two reviews that examined prevalence, identification or intervention for hearing loss, only one was of high quality and neither included data from the regions with the highest prevalence. Infants with universal newborn hearing screening (UNHS) demonstrated a significantly elevated relative risk (RR) of identifying permanent bilateral hearing loss (PBHL) before 9 months, along with an average 13.2 months earlier age of PBHL identification compared to those without UNHS [23].

### Attention deficit/hyperactivity disorder (ADHD)

Globally, approximately 1.4 million (95% UI 898,677–1,947,054) children under 5 years were affected by ADHD in 2019, and half of this cohort was situated within the regions of East Asia (0.5 million) and South Asia (0.2 million) [21]. There were no studies that examined prevention or early detection. The one review that examined early intervention included data from China, Iran and the USA which rank within the top ten highest prevalence countries [37]. The review determined that neurocognitive and behavioural interventions resulted in reduced ADHD symptoms and a positive effect on working memory.

### Autism spectrum disorder (ASD)

Globally, the burden of ASD was estimated to be nearly 3 million (95%UI 2,418,074–3,461,585) children, with Sub-Saharan Africa accounting for approximately 0.8 million cases and the East Asia and Pacific region contributing 0.7 million cases each [21]. Amongst the seven moderate- to high-quality reviews that examined early identification and intervention for ASD, none included data from sub-Saharan Africa, the region with the highest burden. There were no studies on prevention of ASD. The review of 18 screening tests for early detection of ASD found that while diagnostic tools were helpful, their sensitivity and specificity varied [36]. Early intervention studies explored diverse approaches to enhance outcomes for children with developmental challenges and ASD. Spoken word interventions improved spoken language outcomes [32], and community-based interventions enhanced adaptive behaviour [33]. Parent-mediated interventions improved communication [34], although this review was of low quality. Intensive behavioural interventions improved adaptive behaviour [35] and behavioural and social communication interventions enhanced reciprocity of social interaction [38]. The Early Start Denver Model also demonstrated a significant effect on ASD symptoms [39], indicating the potential of these approaches in addressing ASD symptoms and improving outcomes.

## Discussion

We summarised findings from 18 systematic and umbrella reviews that explored evidence-based prevention, early detection, early intervention and rehabilitation for 1,273,444 children with or at risk of developmental disabilities from 251 studies in 30 countries. The majority of reviews (*n* = 12, 67%) focussed on evidence for early intervention. Half of the reviews (*n* = 9) focussed on behavioural disorders, with six (33%) focused on evidence for motor impairment such as cerebral palsy and developmental coordination disorder, and only two reviews (11%) targeted children with hearing impairment. The fewest number of studies were identified for children with cognitive impairment (*n* = 1). Of the 30 countries represented, 20 were high-income countries (67%), ten were middle-income countries (33%) and none were from low-income countries where the prevalence of developmental disabilities was frequently highest. The quality of included reviews was predominantly medium and high.

The synthesis of reviews on prevention for CP highlights the efficacy of interventions such as antenatal corticosteroids [26], magnesium sulfate [26], prophylactic caffeine [27] and neonatal therapeutic hypothermia [27] in reducing CP rates; additionally, early developmental interventions post hospital discharge [28] and environmental enrichment [26] demonstrate promising outcomes in enhancing motor skills and cognitive development for children under five. Moreover, cognitive impairment prevention in preterm infants found that prophylactic use of erythropoietin (rhEPO) [29] demonstrated a significant risk reduction, from 20 to 14%. With regard to hearing impairment, findings suggest that early hearing screening interventions, specifically UNHS, are associated with improved outcomes in identifying hearing loss in infants [23]. There were no meta-analyses for screening for vision, learning disabilities or epilepsy. Regarding ADHD, neurocognitive and behavioural interventions may reduce ADHD symptoms and positively influence working memory [37]. The findings suggest that diagnostic tools for ASD can be useful in early detection, but each test may have varying levels of sensitivity and specificity [36]. Early intervention studies encompassed a range of strategies aimed at enhancing outcomes for children with developmental challenges and ASD, including interventions focusing on improving adaptive behaviour [33, 35], enhancing communication [32, 34] and social interaction [38] and reducing ASD symptoms [31, 39].

The results of this review highlight the disparity between high-income countries and LMICs in terms of evidence availability and applicability to different settings. We identified geographical and disability-related inequities. There is a lack of evidence from outside high-income settings. There was also an absence of data specifically for children with vision loss, even though at least 6 million children under five around the world have a vision impairment [62]. There are also large gaps in early detection. In addition, no developmental screenings during well-child visits were identified in our study. Efforts are therefore needed to gather more data on interventions in LMIC disaggregated by disability type, as this information is crucial to tailoring targeted and appropriate prevention, early detection and rehabilitation interventions.

Our study findings have implications for research. To address study quality, meta-analyses should include an explicit statement regarding the establishment of review methods before conducting the review, a list detailing excluded studies and justifying these exclusions, and investigate publication bias. More generally, there is a lack of data on children under five. Disaggregation by age group and studies that specifically target this age group to inform early interventions are required. Bolstering disability research capabilities across diverse settings is vital to tackle the challenges faced by children with and at risk of developmental disabilities and their caregivers worldwide. Inclusive research practices should emphasise representation and active engagement of children with disabilities and their caregivers to ensure pertinent, considerate and all-encompassing research outcomes.

Our results carry policy and practice implications. We expose gaps in evidence for prevention, identification and early intervention and rehabilitation, along with a disparity between evidence and regions with high prevalence. This underscores the absence of essential evidence for effective strategies in settings with the greatest burden. Importantly, this matter is even more urgent because global financing for rehabilitation, disability and assistive technology is largely not health-led amongst international agencies. A historical emphasis on combatting infectious diseases within the framework of development assistance for health (DAH) has created structures that disenfranchise other health needs—like those of children with disabilities—from core leadership and resources in the sector, including complementary programming. The principal contributor to DAH, the USA [63], largely directs disability-inclusive health investments away from the Global Health Bureau at the United States Agency for International Development (USAID), instead focussing on disproportionately small investments for rehabilitation through its Democracy, Human Rights and Governance sector [64]. It is therefore crucial to align funding strategies with the principles set forth in the Paris Declaration on Aid Effectiveness (2005) [65], including locally led health assistance and prioritisation of health system development, to bridge these disparities and ensure equitable access to appropriate care and interventions for all children. In addition, while the current included reviews have contributed valuable insights into prevalence, interventions and regional disparities, our examination reveals an opportunity for future research to explicitly focus on innovative strategies that challenge societal norms, promote inclusivity and foster a transformative shift in addressing stigma and discrimination associated with developmental disabilities in early childhood.

Supporting all children with disabilities will not be possible without a focus on the integration of evidence-based interventions, inclusive health systems and comprehensive education programmes that prioritise equity, empowerment and inclusion. Access to comprehensive care and support for children with disabilities is crucial for their well-being and overall development. This requires establishing inclusive child health services that cater to diverse needs. By harmonising evidence-based interventions within existing health systems, we can create sustainable and scalable solutions that benefit a larger population.

Further exploration of the interaction between current Early Childhood Development (ECD) programmes and disability support is required. It is evident that many ECD programmes often exclude children with disabilities, which is a missed opportunity for promoting disability-inclusive health and education [3]. However, these ECD initiatives can serve as potential platforms for promoting inclusivity and providing early support to children with disabilities. Finding effective ways to bridge the gap between ECD programmes and disability support could lead to better outcomes and more comprehensive care for all children, regardless of their abilities. This also raises the question of competing agendas, particularly between the focus on human capital development in ECD and the promotion of human rights for children with disabilities. ECD initiatives are often driven by a human capital approach, seeking to enhance children’s skills and abilities for future economic productivity. However, this approach might inadvertently leave behind children with disabilities, as their needs might not align with the productivity-driven goals of human capital development. It is crucial to find a harmonious way to integrate ECD goals with disability rights perspectives, ensuring that all children, including those with disabilities, receive the support they need to thrive and reach their full potential. This integration will require thoughtful policy and programme design, acknowledging and addressing the unique challenges faced by children with disabilities while promoting inclusivity and equity in early childhood development initiatives.

## Strengths and limitations

This paper fills an important gap in the literature with a focus on high burden settings, which previous reviews have lacked. Strengths of this umbrella review include its adherence to standardised guidelines for conducting umbrella reviews and quality assessment, such as following the Preferred Reporting Items for Overviews of Reviews (PRIOR) statement and the AMSTAR2 tool, which has provided methodological rigour, transparency and replicability. The comprehensive search of electronic databases, including relevant broad keywords, helped ensure that a wide range of relevant systematic reviews was identified from 30 countries. Data extraction and quality assessment were conducted independently by two reviewers, reducing bias and enhancing the reliability of the findings. However, there are also limitations to consider. Despite the comprehensive search, it is possible that some relevant systematic reviews might have been missed, particularly as broad search terms were used. For example, parenting interventions. A limitation of the data about ADHD may have arisen from variations in age criteria across settings, where some countries adhere to a lower age cut-off of 4 or 5 years, while the DSM-5 lacks a specified lower age limit, which may potentially result in a lower number of articles available for analysis. Additionally, the absence of disaggregated data for this specific age group poses an issue, potentially resulting in overlooked interventions targeting a broader age range. The decision to exclude certain types of interventions and outcomes, such as surgical interventions and invasive medical procedures that require hospitalisation, may limit the scope of the findings and not fully capture the entire range of interventions available for developmental disabilities.

## Conclusions

This paper summarises the evidence base on effective strategies for prevention, detection and early intervention and rehabilitation for children under 5 years with developmental disabilities globally. We identify a disparity between the settings from which this evidence base comes and the regions where the prevalence is highest. By highlighting the geographical inequities in evidence, we aim to foster a conversation on the allocation of resources and the direction of future research and interventions. Ultimately, this holistic approach has the potential to improve the lives of children with developmental disabilities and their families globally.

### Supplementary Information

**Additional file 1.** Excluded texts, with reasons for exclusion.

**Additional file 2.** Quality assessment of studies, a summary of findings from the quality assessment of selected studies using AMSTAR 2.

**Additional file 3.** Study characteristics of reviews with nonaggregate data, for children of any age.

## Data Availability

All data generated or analysed during this study are included in this published article and its supplementary information files.

## Abbreviations

ADHD: Attention deficit/hyperactivity disorder
AMSTAR: Assessment of Multiple Systematic Reviews
ASD: Autism spectrum disorder
CP: Cerebral palsy
GBD: Global Burden of Disease
LMIC: Low- and middle-income countries
PBHL: Permanent bilateral hearing loss
RR: Risk ratio
SDGs: Sustainable Development Goals
UNHS: Universal newborn hearing screening
