# Supplementary material for: Strategies for addressing the needs of children with or at risk of developmental disabilities in early childhood by 2030: a systematic umbrella review
Source: BMC Med. 2024 Feb 2;22:51. doi: 10.1186/s12916-024-03265-7 (PMC10835858; doi:10.1186/s12916-024-03265-7)
Supplement: Supplementary file 1 — Additional file 1. Excluded texts, with reasons for exclusion. [file 12916_2024_3265_MOESM1_ESM.docx]

**Additional file 1: Excluded texts**

|  | Authors | Title | Notes |
| --- | --- | --- | --- |
| 1 | Abisheva, Y. and Rusetsky, Y. and Daniyarova, A. and Azhenov, T. and Imasheva, B. and Almabayev, Y. and Turysbekova, D. and Utegenov, A. | Application of IT technology in the management of voice-speech disorders and phoniatric rehabilitation | Conference abstract, No meta-analysis |
| 2 | Acar, S. and Chen, C. I. and Xie, H. | Parental involvement in developmental disabilities across three cultures: A systematic review | No meta-analysis |
| 3 | Adair, B. and Ullenhag, A. and Keen, D. and Granlund, M. and Imms, C. | The effect of interventions aimed at improving participation outcomes for children with disabilities: A systematic review | No meta-analysis |
| 4 | Agyeman-Duah, J. and Kennedy, S. and O'Brien, F. and Natalucci, G. | Interventions to improve neurodevelopmental outcomes of children born moderate to late preterm: A systematic review protocol | Protocol |
| 5 | Alfiyanti, D. and Pandin, M. G. R. and Rahayuningtyas, I. R. | A literature review on complementary therapies in autism children: a nursing science philosophy | No meta-analysis |
| 6 | Alonso-Esteban, Yurena and Marco, Rafaela and Hedley, Darren and Uljarevie, Mirko and Barbaro, Josie and Canal-Bedia, Ricardo and Alcantud-Marin, Francisco | Screening instruments for early detection of autism spectrum disorder in Spanish speaking communities | No meta-analysis |
| 7 | Alrashidi, M. and Wadey, C. A. and Tomlinson, R. J. and Buckingham, G. and Williams, C. A. | The efficacy of virtual reality interventions compared with conventional physiotherapy in improving the upper limb motor function of children with cerebral palsy: a systematic review of randomised controlled trials | No meta-analysis |
| 8 | Alvito, P. and Pereira-Da-silva, L. | Mycotoxin Exposure during the First 1000 Days of Life and Its Impact on Children's Health: A Clinical Overview | No meta-analysis, No intervention |
| 9 | Anonymous | Early treatment of premature infants with steroids: Neurological sequelae | No meta-analysis, No full text |
| 10 | Anonymous | American Academy for Cerebral Palsy and Developmental Medicine 64th Annual Meeting | No meta-analysis, Conference abstract |
| 11 | Anonymous | Abstracts of the European Academy of Childhood Disability 26th Annual Meeting | No meta-analysis, Conference abstract |
| 12 | Anonymous | Abstracts for the Australasian Academy of Cerebral Palsy and Developmental Medicine 2018 | No meta-analysis, Conference abstract |
| 13 | Anonymous | Abstracts of the 33rd Annual Meeting of the European Academy of Childhood Disability, EACD | No meta-analysis, Conference abstract |
| 14 | Anonymous | Australasian Academy of Cerebral Palsy and Developmental Medicine and the International Alliance of Academies of Childhood Disability Conference | No meta-analysis, Conference abstract |
| 15 | Appachi, S. and Specht, J. L. and Raol, N. and Lieu, J. E. C. and Cohen, M. S. and Dedhia, K. and Anne, S. | Auditory Outcomes with Hearing Rehabilitation in Children with Unilateral Hearing Loss: A Systematic Review | No meta-analysis |
| 16 | Asta, L. and Persico, A.M. | Differential Predictors of Response to Early Start Denver Model vs. Early Intensive Behavioral Intervention in Young Children with Autism Spectrum Disorder: A Systematic Review  and Meta-Analysis | Wrong outcome |
| 17 | Auld, M. L. and Russo, R. and Moseley, G. L. and Johnston, L. M. | Determination of interventions for upper extremity tactile impairment in children with cerebral palsy: A systematic review | No meta-analysis |
| 18 | Behrendt, F. and Zumbrunnen, V. and Brem, L. and Suica, Z. and Gaumann, S. and Ziller, C. and Gerth, U. and Schuster-Amft, C. | Effect of motor imagery training on motor learning in children and adolescents: A systematic review and meta-analysis | No meta-analysis |
| 19 | Birch, E. E. and Kelly, K. R. and Wang, J. | Recent Advances in Screening and Treatment for Amblyopia | No meta-analysis |
| 20 | Bitsko, R. H. and Holbrook, J. R. and O'Masta, B. and Maher, B. and Cerles, A. and Saadeh, K. and Mahmooth, Z. and MacMillan, L. M. and Rush, M. and Kaminski, J. W. | A Systematic Review and Meta-analysis of Prenatal, Birth, and Postnatal Factors Associated with Attention-Deficit/Hyperactivity Disorder in Children | No intervention |
| 21 | Blencowe, Hannah and Kancherla, Vijaya and Moorthie, Sowmiya and Darlison, Matthew W. and Modell, Bernadette | Estimates of global and regional prevalence of neural tube defects for 2015: a systematic analysis | No intervention, no intervention |
| 22 | Bleyenheuft, Yannick and Gordon, Andrew M. | Precision grip control, sensory impairments and their interactions in children with hemiplegic cerebral palsy: a systematic review | No meta-analysis |
| 23 | Blumetti, F. C. and Belloti, J. C. and Tamaoki, M. J. S. and Pinto, J. A. | Botulinum toxin type A in the treatment of lower limb spasticity in children with cerebral palsy | No meta-analysis |
| 24 | Boster, Jamie B. and Spitzley, Alyson M. and Castle, Taylor W. and Jewell, Abby R. and Corso, Christina L. and McCarthy, John W. | Music Improves Social and Participation Outcomes for Individuals With Communication Disorders: A Systematic Review | No meta-analysis |
| 25 | Bowman-Perrott, L. and Gilson, C. and Boon, R. T. and Ingles, K. E. | Peer-Mediated Interventions for Students with Intellectual and Developmental Disabilities: A Systematic Review of Reviews of Social and Behavioral Outcomes | No meta-analysis |
| 26 | Brignell, A. and Harwood, R. C. and May, T. and Woolfenden, S. and Montgomery, A. and Iorio, A. and Williams, K. | Overall prognosis of preschool autism spectrum disorder diagnoses | Prevalence study |
| 27 | Bruschettini, M. and Romantsik, O. and Moreira, A. and Ley, D. and ThÃ©baud, B. | Stem cell based interventions for the prevention of morbidity and mortality following hypoxic ischaemic encephalopathy in newborn infants | No meta-analysis |
| 28 | Byrne, Rachel and Noritz, Garey and Maitre, Nathalie L. | Implementation of Early Diagnosis and Intervention Guidelines for Cerebral Palsy in a High-Risk Infant Follow-Up Clinic | No meta-analysis, Not a systematic review |
| 29 | Calder, Samuel and Ward, Roslyn and Jones, Megan and Johnston, Jenelle and Claessen, Mary | The uses of outcome measures within multidisciplinary early childhood intervention services: a systematic review | No meta-analysis |
| 30 | Camden, Chantal and Pratte, Gabrielle and Fallon, Florence and Couture, Melanie and Berbari, Jade and Tousignant, Michel | Diversity of practices in telerehabilitation for children with disabilities and effective intervention characteristics: Results from a systematic review | No meta-analysis |
| 31 | Casoojee, A. and Kanji, A. and Khoza-Shangase, K. | Therapeutic approaches to early intervention in audiology: A systematic review | No meta-analysis |
| 32 | Cavalli, G. and Galeoto, G. and Sogos, C. and Berardi, A. and Tofani, M. | The efficacy of executive function interventions in children with autism spectrum disorder: a systematic review and meta-analysis | No meta-analysis |
| 33 | Chennariyil, L. and Williams, K. and Bayl, V. and Sarkozy, V. | Prognosis of children with idiopathic developmental delay seen in a tertiary developmental diagnostic and assessment service | No meta-analysis, No full text |
| 34 | Clairmont, Cullen and Wang, Jiuju and Tariq, Samia and Sherman, Hannah Tayla and Zhao, Mingxuan and Kong, Xue-Jun | The Value of Brain Imaging and Electrophysiological Testing for Early Screening of Autism Spectrum Disorder: A Systematic Review | No meta-analysis |
| 35 | Colombani, A. and Saksida, A. and Pavani, F. and Orzan, E. | Symbolic and deictic gestures as a tool to promote parent-child communication in the context of hearing loss: A systematic review | No meta-analysis |
| 36 | Colomera, J. A. and Nahuelhual, P. | [Effectiveness of robotic assistance for gait training in children with cerebral palsy. a systematic review] | No meta-analysis |
| 37 | Cumplido, Carlos and Delgado, Elena and Ramos, Jaime and Puyuelo, Gonzalo and Garces, Elena and Destarac, Marie Andre and Plaza, Alberto and Hernandez, Mar and Gutierrez, Alba and Garcia, Elena | Gait-assisted exoskeletons for children with cerebral palsy or spinal muscular atrophy: A systematic review | No meta-analysis |
| 38 | Cunha, Andrea Baraldi and Lima-Alvarez, Carolina Daniel de and Rocha, Ana Carolinne Portela and Tudella, Eloisa | Effects of elastic therapeutic taping on motor function in children with motor impairments: a systematic review | No meta-analysis |
| 39 | Dahiya, Angela V. and McDonnell, Christina and DeLucia, Elizabeth and Scarpa, Angela | A systematic review of remote telehealth assessments for early signs of autism spectrum disorder: Video and mobile applications | No meta-analysis |
| 40 | Damiano, Diane L. and DeJong, Stacey L. | A systematic review of the effectiveness of treadmill training and body weight support in pediatric rehabilitation | No meta-analysis |
| 41 | den Brok, W. L. J. E. and Sterkenburg, P. S. | Self-controlled technologies to support skill attainment in persons with an autism spectrum disorder and/or an intellectual disability: a systematic literature review | No meta-analysis |
| 42 | Denver, B. | Development and initial validation of an assessment of visual ability for children with cerebral palsy | No meta-analysis, Not peer-reviewed |
| 43 | du Toit, R. and Courtright, P. and Lewallen, S. | The Use of Key Informant Method for Identifying Children with Blindness and Severe Visual Impairment in Developing Countries | No meta-analysis |
| 44 | Dubin, Ashley H. and Lieberman-Betz, Rebecca G. | Naturalistic interventions to improve prelinguistic communication for children with autism spectrum disorder: A systematic review | No meta-analysis |
| 45 | Fernandes, Romita and Hariprasad, Shalini and Kumar, Vijaya K. | Physical therapy management for balance deficits in children with hearing impairments: A systematic review | No meta-analysis |
| 46 | Ferre Rey, Gisela and Sanchez Rodriguez, Josefina and Llorca Linares, Miguel and Vicens, Paloma and Camps, Misericordia and Torrente, Margarita and Morales Vives, Fabia | A systematic review of instruments for early detection of autism spectrum disorders | No meta-analysis |
| 47 | Franki, I. and Van Baelen, A. and Itzhak, N. and Ortibus, E. | Lessons learned regarding the effectiveness of different visual perceptual training methods: A systematic review | No meta-analysis, No full text |
| 48 | Freitag, Christine M. and Jensen, Katrin and Teufel, Karoline and Luh, Marvin and Todorova, Antoaneta and Lalk, Christopher and Vllasaliu, Leonora | Empirically based developmental and behavioral intervention programs targeting the core symptoms and language development in toddlers and preschool children with autism spectrum disorder | No meta-analysis |
| 49 | French, Lorna and Kennedy, Eilis M. M. | Annual research review: Early intervention for infants and young children with, or at-risk of, autism spectrum disorder: A systematic review | No meta-analysis |
| 50 | Frizelle, P. and Tolonen, A. K. and Tulip, J. and Murphy, C. A. and Saldana, D. and McKean, C. | The Impact of Intervention Dose Form on Oral Language Outcomes for Children With Developmental Language Disorder | No meta-analysis |
| 51 | Froguel, P. | Harnessing our knowledge of human genetics in the prevention and treatment of childhood obesity | No full text, wrong population |
| 52 | Frosolini, A. and Fantin, F. and Tundo, I. and Pessot, N. and Badin, G. and Bartolotta, P. and Vedovelli, L. and Marioni, G. and de Filippis, C. | Voice Parameters in Children With Cochlear Implants: A Systematic Review and Meta-Analysis | No intervention |
| 53 | Fu, W. and Ji, C. | Application and Effect of Virtual Reality Technology in Motor Skill Intervention for Individuals with Developmental Disabilities: A Systematic Review | No meta-analysis |
| 54 | Garcia, Rafael Ferro and Bocanegra, Maria Rodriguez and Velasco, Lourdes Ascanio | A systematic review of the effectiveness and efficacy of parent-child interaction therapy | No meta-analysis |
| 55 | Gargot, T. | Diagnostic automated algorithms in neurodevelopmental disorders: Focus on automatic motor assessment | No meta-analysis, Conference abstract |
| 56 | Gargot, T. | Sensory and Motor Difficulties in Autism | No meta-analysis, Conference abstract |
| 57 | Ghasemifard, F. and Mirzaie, H. and Oori, M. J. and Riazi, A. | Characteristics and efficacy of play therapy interventions in visually impaired children and adolescents: A systematic review study | No meta-analysis |
| 58 | Guchan Topcu, Zehra and Tomac, Hayriye | The Effectiveness of Massage for Children With Cerebral Palsy: A Systematic Review | No meta-analysis |
| 59 | Harding, Katherine E. and Camden, Chantal and Lewis, Annie K. and Perreault, Kadija and Taylor, Nicholas F. | Service redesign interventions to reduce waiting time for paediatric rehabilitation and therapy services: A systematic review of the literature | No meta-analysis |
| 60 | Harris, Emma and Samuel, Victoria | Acceptance and commitment therapy: A systematic literature review of prevention and intervention programs for mental health difficulties in children and young people | No meta-analysis |
| 61 | Heidlage, Jodi K. and Cunningham, Jennifer E. and Kaiser, Ann P. and Trivette, Carol M. and Barton, Erin E. and Frey, Jennifer R. and Roberts, Megan Y. | The effects of parent-implemented language interventions on child linguistic outcomes: A meta-analysis | Not child with disability |
| 62 | Heydarian, Samira and Abbasabadi, Marziye Moradi and Khabazkhoob, Mehdi and Hoseini-Yazdi, Hosein and Gharib, Masoud | Vision Abnormalities in Children and Young Adults With Cerebral Palsy; A Systematic Review | Wrong outcome |
| 63 | Hiremath, C. S. and Sagar, K. J. V. and Yamini, B. K. and Girimaji, A. S. and Kumar, R. and Sravanti, S. L. and Padmanabha, H. and Vykunta Raju, K. N. and Kishore, M. T. and Jacob, P. and Saini, J. and Bharath, R. D. and Seshadri, S. P. and Kumar, M. | Emerging behavioral and neuroimaging biomarkers for early and accurate characterization of autism spectrum disorders: a systematic review | No meta-analysis |
| 64 | Huang, Chuyao and Chen, Yijun and Chen, Guoming and Xie, Yaying and Mo, Jiahao and Li, Keyi and Huang, RuiLan and Pan, Guanghua and Cai, Yong and Zhou, Lei | Efficacy and safety of core stability training on gait of children with cerebral palsy: A protocol for a systematic review and meta-analysis | Protocol |
| 65 | Izadi-Najafabadi, Sara and Rinat, Shie and Zwicker, Jill G. | Rehabilitation-induced brain changes detected through magnetic resonance imaging in children with neurodevelopmental disorders: A systematic review | No meta-analysis |
| 66 | Jamali, A. R. and Amini, M. | The effects of constraint induced movement therapy on functions of children with cerebral palsy | No meta-analysis |
| 67 | Jardine, J. and Glinianaia, S. V. and McConachie, H. and Embleton, N. D. and Rankin, J. | Self-reported quality of life of young children with conditions from early infancy: A systematic review | No meta-analysis |
| 68 | Joseph, H. M. and Lorenzo, N. E. and Fisher, N. and Novick, D. R. and Gibson, C. and Rothenberger, S. D. and Foust, J. E. and Chronis-Tuscano, A. | Research Review: A systematic review and meta-analysis of infant and toddler temperament as predictors of childhood attention-deficit/hyperactivity disorder | No intervention |
| 69 | Keeratisiroj, Orawan and Thawinchai, Nuanlaor and Siritaratiwat, Wantana and Buntragulpoontawee, Montana and Pratoomsoot, Chayanin | Prognostic predictors for ambulation in children with cerebral palsy: a systematic review and meta-analysis of observational studies | No intervention |
| 70 | Kiling, Indra Yohanes and Due, Clemence and Gyss, Cameron and Li, Dominggus Elcid and Turnbull, Deborah | Intervention research addressing environmental risk threatening young children with disabilities in developing countries: a systematic review | No meta-analysis |
| 71 | King, Alison and Xu, Yaoying | Caregiver coaching for language facilitation in early intervention for children with hearing loss | No meta-analysis |
| 72 | Klasen, H. | What works where? A systematic review of child and adolescent mental health interventions for low and middle-income countries | No meta-analysis |
| 73 | Kokol, P. and Vosner, H. B. and Zavrsnik, J. and Vermeulen, J. and Shohieb, S. and Peinemann, F. | Serious game-based intervention for children with developmental disabilities | No meta-analysis, Conference abstract |
| 74 | Lee, B. and Kwon, C. Y. and Chang, G. T. | Oriental herbal medicine for neurological disorders in children: An overview of systematic reviews | No meta-analysis |
| 75 | Lefmann, S. and Russo, R. and Hillier, S. | What evidence exists on the effectiveness of the use of robotic-assisted gait training in children with neurological gait disorders? | Excluded age range |
| 76 | Liang, X. and Li, R. and Wong, S. H. S. and Sum, R. K. W. and Sit, C. H. P. | The impact of exercise interventions concerning executive functions of children and adolescents with attention-deficit/hyperactive disorder: a systematic review and meta-analysis | Excluded age range |
| 77 | Lima, E. and Brugnaro, B. and Pavao, S. and Rocha, N. | Effects of Dance Interventions on Functioning of Children and Adolescents with Neuromotor Dysfunction: A Systematic Review | No meta-analysis |
| 78 | Lobato Garcia, L. and Gonzalez Gonzalez, Y. and Da Cuna Carrera, I. and Alonso Calvete, A. | [Benefits of robotics in gait rehabilitation in cerebral palsy: A systematic review] | No meta-analysis |
| 79 | Lopes, Jamile Benite Palma and Duarte, Natalia de Almeida Carvalho and Lazzari, Roberta Delasta and Oliveira, Claudia Santos | Virtual reality in the rehabilitation process for individuals with cerebral palsy and Down syndrome: A systematic review | No meta-analysis |
| 80 | Lynam, Aideen and Smith, Martine M. | Sibling involvement in interventions for children with a disability: a systematic review | No meta-analysis |
| 81 | Magalhaes, L. C. and Cardoso, A. A. and Missiuna, C. | Activities and participation in children with developmental coordination disorder: A systematic review | No meta-analysis |
| 82 | Marchi, A. R. | The importance of early intensive behavioural intervention in autism spectrum disorder | No meta-analysis |
| 83 | Masuda, F. and Nakajima, S. and Miyazaki, T. and Tarumi, R. and Ogyu, K. and Wada, M. and Tsugawa, S. and Croarkin, P. E. and Mimura, M. and Noda, Y. | Clinical effectiveness of repetitive transcranial magnetic stimulation treatment in children and adolescents with neurodevelopmental disorders: A systematic review | No meta-analysis |
| 84 | Mayoâ€Wilson, E. and Montgomery, P. and Dennis, J. A. | Personal assistance for children and adolescents (0â€18) with physical impairments | No meta-analysis |
| 85 | McCarthy, Elaine and Guerin, Suzanne | Family-centred care in early intervention: A systematic review of the processes and outcomes of family-centred care and impacting factors | No meta-analysis |
| 86 | Micai, M. and Fulceri, F. and Caruso, A. and Guzzetta, A. and Gila, L. and Scattoni, M. L. | Early behavioral markers for neurodevelopmental disorders in the first 3 years of life: An overview of systematic reviews | No intervention |
| 87 | Mirkowski, M. and Cotoi, A. and McIntyre, A. and Sequeira, N. and Cassidy, C. and Teasell, R. | Nonpharmacological rehabilitation interventions for motor and cognitive outcomes following paediatric stroke: A systematic review | No meta-analysis |
| 88 | Mitchell, L. and Ziviani, J. and Oftedal, S. and Boyd, R. | Efficacy of virtual-reality interventions to increase physical activity in children and adolescents with cerebral palsy: A systematic review | No meta-analysis, Excluded age range |
| 89 | Miyahara, M. and Hillier, S. L. and Pridham, L. and Nakagawa, S. | Task-oriented interventions for children with developmental co-ordination disorder | Excluded age range |
| 90 | Morgan, C. and Fetters, L. and Adde, L. and Badawi, N. and Bancale, A. and Boyd, R. N. and Chorna, O. and Cioni, G. and Damiano, D. L. and Darrah, J. and De Vries, L. S. and Dusing, S. and Einspieler, C. and Eliasson, A. C. and Ferriero, D. and Fehlings, D. and Forssberg, H. and Gordon, A. M. and Greaves, S. and Guzzetta, A. and Hadders-Algra, M. and Harbourne, R. and Karlsson, P. and Krumlinde-Sundholm, L. and Latal, B. and Loughran-Fowlds, A. and Mak, C. and Maitre, N. and McIntyre, S. and Mei, C. and Morgan, A. and Kakooza-Mwesige, A. and Romeo, D. M. and Sanchez, K. and Spittle, A. and Shepherd, R. and Thornton, M. and Valentine, J. and Ward, R. and Whittingham, K. and Zamany, A. and Novak, I. | Early Intervention for Children Aged 0 to 2 Years with or at High Risk of Cerebral Palsy: International Clinical Practice Guideline Based on Systematic Reviews | No meta-analysis |
| 91 | Mulligan, H. and Rowland, J. L. and Sandlund, M. and Potterton, J. and Kanagasabai, P. | Emerging use of interactive technology in rehabilitation for young people | No meta-analysis, Conference abstract |
| 92 | Murugasen, S. and Springer, P. and Donald, K. A. | A systematic review of cerebral palsy in African paediatric populations | No meta-analysis, Not peer-reviewed |
| 93 | Noel, Augustina and Manikandan, Manju and Kumar, Prawin | Efficacy of auditory verbal therapy in children with cochlear implantation based on auditory performance - A systematic review | no intervention |
| 94 | Novak, I. | Effectiveness of occupational therapy intervention for children with disabilities: Systematic review | No meta-analysis |
| 95 | Novak, I. and Morgan, C. and Adde, L. and Blackman, J. and Boyd, R. N. and Brunstrom-Hernandez, J. and Cioni, G. and Damiano, D. and Darrah, J. and Eliasson, A. C. and De Vries, L. S. and Einspieler, C. and Fahey, M. and Fehlings, D. and Ferriero, D. M. and Fetters, L. and Fiori, S. and Forssberg, H. and Gordon, A. M. and Greaves, S. and Guzzetta, A. and Hadders-Algra, M. and Harbourne, R. and Kakooza-Mwesige, A. and Karlsson, P. and Krumlinde-Sundholm, L. and Latal, B. and Loughran-Fowlds, A. and Maitre, N. and McIntyre, S. and Noritz, G. and Pennington, L. and Romeo, D. M. and Shepherd, R. and Spittle, A. J. and Thornton, M. and Valentine, J. and Walker, K. and White, R. and Badawi, N. | Early, accurate diagnosis and early intervention in cerebral palsy: Advances in diagnosis and treatment | No meta-analysis |
| 96 | Novak, I. and Morgan, C. and Fahey, M. and Finch-Edmondson, M. and Galea, C. and Hines, A. and Langdon, K. and Namara, M. M. and Paton, M. C. and Popat, H. and Shore, B. and Khamis, A. and Stanton, E. and Finemore, O. P. and Tricks, A. and te Velde, A. and Dark, L. and Morton, N. and Badawi, N. | State of the Evidence Traffic Lights 2019: Systematic Review of Interventions for Preventing and Treating Children with Cerebral Palsy | Repeat |
| 97 | Ogourtsova, Tatiana and Boychuck, Zachary and O'Donnell, Maureen and Ahmed, Sara and Osman, Galil and Majnemer, Annette | Telerehabilitation for Children and Youth with Developmental Disabilities and Their Families: A Systematic Review | No meta-analysis |
| 98 | Oliva, F. and Malandrone, F. and di Girolamo, G. and Mirabella, S. and Colombi, N. and Carletto, S. and Ostacoli, L. | The efficacy of mindfulness-based interventions in attention-deficit/hyperactivity disorder beyond core symptoms: A systematic review, meta-analysis, and meta-regression | Children 7-16 |
| 99 | Olusanya, Bolajoko O. and Somefun, Abayomi O. and Swanepoel, De Wet | The need for standardization of methods for worldwide infant hearing screening: a systematic review | No meta-analysis |
| 100 | Papacharalampous, G. X. and Nikolopoulos, T. P. and Davilis, D. I. and Xenellis, I. E. and Korres, S. G. | Universal newborn hearing screening, a revolutionary diagnosis of deafness: Real benefits and limitations | No meta-analysis |
| 101 | Pennington, L. and Akor, W. A. and Laws, K. and Goldbart, J. | Parental mediated communication interventions for improving the communication skills of preschool children with non progressive motor disorders | No meta-analysis |
| 102 | Peterson, B. and Frias, J. and Tourgeman, I. | Examining Cogmed's Impact on the Cognitive Improvement of Individuals with Traumatic Brain Injury | No meta-analysis |
| 103 | Pimentel-Ponce, M. and Romero-Galisteo, R. P. and Palomo-Carrion, R. and Pinero-Pinto, E. and Merchan-Baeza, J. A. and Ruiz-Munoz, M. and Oliver-Pece, J. and Gonzalez-Sanchez, M. | Gamification and neurological motor rehabilitation in children and adolescents: A systematic review | No meta-analysis |
| 104 | Premji, S. and Benzies, K. and Serrett, K. and Hayden, K. A. | Research-based interventions for children and youth with a Fetal Alcohol Spectrum Disorder: Revealing the gap | No meta-analysis |
| 105 | Ragni, B. and Boldrini, F. and Mangialavori, S. and Cacioppo, M. and Capurso, M. and De Stasio, S. | The Efficacy of Parent Training Interventions with Parents of Children with Developmental Disabilities | No meta-analysis |
| 106 | Rastogi, S. and Stinson, J. and Campbell, F. and Hajee, S. | Narrative systematic review of interdisciplinary rehabilitation programs for children and adolescents with chronic pain | No meta-analysis |
| 107 | Rastogi, S. and Stinson, J. and Campbell, F. and Hajee, S. and Kazazian, V. | Rehabilitation programs pediatric chronic pain: Systematic review | No meta-analysis |
| 108 | Ravi, D. K. and Kumar, N. and Singhi, P. | Effectiveness of virtual reality rehabilitation for children and adolescents with cerebral palsy: an updated evidence-based systematic review | No meta-analysis |
| 109 | Ravi, Rohit and Gunjawate, Dhanshree R. | Parent reported barriers and facilitators towards cochlear implantation - A systematic review | No meta-analysis |
| 110 | Reichow, Brian and Servili, Chiara and Yasamy, M. Taghi and Barbui, Corrado and Saxena, Shekhar | Non-specialist psychosocial interventions for children and adolescents with intellectual disability or lower-functioning autism spectrum disorders: a systematic review | No meta-analysis |
| 111 | Reid, N. and Dawe, S. and Shelton, D. and Harnett, P. and Warner, J. and Armstrong, E. and Legros, K. and O'Callaghan, F. | Systematic Review of Fetal Alcohol Spectrum Disorder Interventions Across the Life Span | No meta-analysis |
| 112 | Retamal-Walter, F. and Waite, M. and Scarinci, N. | Exploring engagement in telepractice early intervention for young children with developmental disability and their families: a qualitative systematic review | No meta-analysis |
| 113 | Reynhout, G. and Carter, M. | Social stories™ for children with disabilities | wrong date |
| 114 | Rezayi, Sorayya and Tehrani-Doost, Mehdi and Shahmoradi, Leila | Features and effects of computer-based games on cognitive impairments in children with autism spectrum disorder: An evidence-based systematic literature review | No meta-analysis |
| 115 | Richard, C. and Kjeldsen, C. and Findlen, U. and Gehred, A. and Maitre, N. L. | Hearing Loss Diagnosis and Early Hearing-Related Interventions in Infants With or at High Risk for Cerebral Palsy: A Systematic Review | No meta-analysis |
| 116 | Rivers, J. and Nye, C. | The conclusion that ABI has inconclusive effects for children with autism may stem from the fact that there are few high quality studies | wrong date |
| 117 | Robles, N. and Carrion, C. and Ribas, I. and Pamias, M. and Parra, I. and Conesa, J. and Perez-Navarro, A. and Alabert, M. and Aymerich, M. | A mobile clinical decision support system for autism spectrum disorder | No meta-analysis |
| 118 | Rodgers, L. and Harding, S. and Rees, R. and Clarke, M. T. | Interventions for pre-school children with co-occurring phonological speech sound disorder and expressive language difficulties: A scoping review | No meta-analysis |
| 119 | Rodrigo-Yanguas, Maria and Gonzalez-Tardon, Carlos and Bella-Fernandez, Marcos and Blasco-Fontecilla, Hilario | Serious Video Games: Angels or Demons in Patients With Attention-Deficit Hyperactivity Disorder? A Quasi-Systematic Review | No meta-analysis |
| 120 | Rodrigues, F. B. and Duarte, G. S. and Prescott, D. and Ferreira, J. and Costa, J. | Deep brain stimulation for dystonia | adults, No meta-analysis |
| 121 | Rodrigues, J. M. and Mestre, M. and Fredes, L. I. | Qigong in the treatment of children with autism spectrum disorder: A systematic review | No meta-analysis |
| 122 | Rohn, S. and Novak Pavlic, M. and Rosenbaum, P. | Exploring the use of Halliwick aquatic therapy in the rehabilitation of children with disabilities: A scoping review | No meta-analysis |
| 123 | Rojas, L. M. and Bahamon, M. and Wagstaff, R. and Ferre, I. and Perrino, T. and Estrada, Y. and St. George, S. M. and Pantin, H. and Prado, G. | Evidence-based prevention programs targeting youth mental and behavioral health in primary care: A systematic review | No meta-analysis |
| 124 | Rueda, J. R. and Ballesteros, J. and Guillen, V. and Tejada, M. I. and SolÃ , I. | Folic acid for fragile X syndrome | Wrong population |
| 125 | Ruggeri, A. and Dancel, A. and Johnson, R. and Sargent, B. | The effect of motor and physical activity intervention on motor outcomes of children with autism spectrum disorder: A systematic review | No meta-analysis |
| 126 | Ryan, J. M. and Cassidy, E. E. and Noorduyn, S. G. and O'Connell, N. E. | Exercise interventions for cerebral palsy | No meta-analysis |
| 127 | Sabir, O. A. and Alshomrani, A. M. and Alqarni, W. M. and Bamusa, K. A. and Johnson, E. G. | Effects of vestibular rehabilitation exercises on children with hearing loss, cerebral palsy, and attention deficit hyperactivity disorder: A systematic review | No meta-analysis |
| 128 | Sampaio, F. and Feldman, I. and Lavelle, T. A. and Skokauskas, N. | The cost-effectiveness of treatments for attention deficit-hyperactivity disorder and autism spectrum disorder in children and adolescents: a systematic review | No meta-analysis |
| 129 | Sanner, J. R. F. and Jain, K. and Williams, J. and Hurley, M. N. | Antibiotics for chronic pulmonary infection in children with a neurodisability (neurodevelopmental disorder) | wrong outcome |
| 130 | Sgherri, G. and Beani, E. and Cioni, G. and Sgandurra, G. | A literature review on therapeutic management of upper limb dysfunction in children with bilateral spastic cerebral palsy | failed to access the full article |
| 131 | Shahmoradi, Leila and Rezayi, Sorayya | Cognitive rehabilitation in people with autism spectrum disorder: a systematic review of emerging virtual reality-based approaches | No meta-analysis |
| 132 | Shahouzaie, N. and Gholamiyan Arefi, M. | Telehealth in speech and language therapy during the COVID-19 pandemic: a systematic review | No meta-analysis |
| 133 | Sherr, Lorraine and Croome, Natasha and Bradshaw, Katie and Parra Castaneda, Katherine | A systematic review examining whether interventions are effective in reducing cognitive delay in children infected and affected with HIV | No meta-analysis |
| 134 | Sibbick, E. and Boat, R. and Sarkar, M. and Groom, M. and Cooper, S. B. | Acute effects of physical activity on cognitive function in children and adolescents with attention-deficit/hyperactivity disorder: A systematic review and meta-analysis | ages 6-16 years |
| 135 | Sissons, J. H. and Blakemore, E. and Shafi, H. and Skotny, N. and Lloyd, D. M. | Calm with horses? A systematic review of animal-assisted interventions for improving social functioning in children with autism | No meta-analysis |
| 136 | Soleimani, M. and Rouhbakhsh, N. and Rahbar, N. | Towards early intervention of hearing instruments using cortical auditory evoked potentials (CAEPs): A systematic review | No meta-analysis |
| 137 | Sommese, M. and Corrado, B. | A comprehensive approach to rehabilitation interventions in patients with angelman syndrome: A systematic review of the literature | wrong population |
| 138 | Symons, M. and Pedruzzi, R. A. and Bruce, K. and Milne, E. | A systematic review of prevention interventions to reduce prenatal alcohol exposure and fetal alcohol spectrum disorder in indigenous communities | No meta-analysis |
| 139 | Tomeny, Kimberly R. and McWilliam, R. A. and Tomeny, Theodore S. | Caregiver-implemented intervention for young children with autism spectrum disorder: A systematic review of coaching components | No meta-analysis |
| 140 | Torra Moreno, Marta and Canals Sans, Josefa and Colomina Fosch, Maria Teresa | Behavioral and cognitive interventions with digital devices in subjects with intellectual disability: A systematic review | No meta-analysis |
| 141 | Townley, A. and Kingsnorth, S. and Orava, T. and Provvidenza, C. | An evidence-informed approach to the identification and assessment of chronic pain in children with cerebral palsy | No meta-analysis |
| 142 | Trevor, Maxfield and Park, Eun-Young and Blair, Kwang-Sun Cho | A meta-analysis of safety skills interventions for individuals with intellectual disabilities | wrong outcome |
| 143 | Tupou, Jessica and van der Meer, Larah and Waddington, Hannah and Sigafoos, Jeff | Preschool interventions for children with autism spectrum disorder: A review of effectiveness studies | No meta-analysis |
| 144 | Twardzik, E. and Cotto-Negron, C. and MacDonald, M. | Factors related to early intervention Part C enrollment: A systematic review | No meta-analysis |
| 145 | Van Cleave, J. and Kuhlthau, K. A. and Bloom, S. and Newacheck, P. W. and Nozzolillo, A. A. and Homer, C. J. and Perrin, J. M. | Interventions to improve screening and follow-up in primary care: A systematic review of the evidence | No meta-analysis |
| 146 | Van der Meer, L. and Sigafoos, J. and O'Reilly, M. F. and Lancioni, G. E. | Assessing preferences for AAC options in communication interventions for individuals with developmental disabilities: A review of the literature | No meta-analysis |
| 147 | Vereenooghe, L. and Flynn, S. and Hastings, R. P. and Adams, D. and Chauhan, U. and Cooper, S. A. and Gore, N. and Hatton, C. and Hood, K. and Jahoda, A. and Langdon, P. E. and McNamara, R. and Oliver, C. and Roy, A. and Totsika, V. and Waite, J. | Interventions for mental health problems in children and adults with severe intellectual disabilities: A systematic review | No meta-analysis |
| 148 | Vodopivecâ€Jamsek, V. and de Jongh, T. and Gurolâ€Urganci, I. and Atun, R. and Car, J. | Mobile phone messaging for preventive health care | adults |
| 149 | Walaszek, R. and Masnik, N. and Marszalek, A. and Walaszek, K. and Burdacki, M. | Massage efficacy in the treatment of autistic children-a literature review | No meta-analysis |
| 150 | Wallace, Sarah and Alao, Rotimi and Kuper, Hannah and Jackson, Mary Lou | Multidisciplinary visual rehabilitation in low- and middle-income countries: A systematic review | No meta-analysis |
| 151 | Wang, J. and Hedley, D. and Bury, S. M. and Barbaro, J. | A systematic review of screening tools for the detection of autism spectrum disorder in mainland China and surrounding regions | No meta-analysis |
| 152 | Ward, Roslyn and Reynolds, Jess E. and Pieterse, Bridget and Elliott, Catherine and Boyd, Roslyn and Miller, Laura | Utilisation of coaching practices in early interventions in children at risk of developmental disability/delay: A systematic review | No meta-analysis |
| 153 | Watkins, L. and O'Reilly, M. and Kuhn, M. and Gevarter, C. and Lancioni, G. E. and Sigafoos, J. and Lang, R. | A Review of Peer-Mediated Social Interaction Interventions for Students with Autism in Inclusive Settings | No meta-analysis |
| 154 | Winfield, N. R. and Barker, N. J. and Turner, E. R. and Quin, G. L. | Nonpharmaceutical management of respiratory morbidity in children with severe global developmental delay | No meta-analysis |
| 155 | Wissow, L. S. and Brown, J. and Fothergill, K. E. and Gadomski, A. and Hacker, K. and Salmon, P. and Zelkowitz, R. | Universal mental health screening in pediatric primary care: A systematic review | No meta-analysis |
| 156 | Wolff, R. and Hommerich, J. and Riemsma, R. and Antes, G. and Lange, S. and Kleijnen, J. | Hearing screening in newborns: systematic review of accuracy, effectiveness, and effects of interventions after screening | No meta-analysis |
| 157 | Xu, G. and Hao, F. and Zhao, W. and Qiu, J. and Zhao, P. and Zhang, Q. | The influential factors and non-pharmacological interventions of cognitive impairment in children with ischemic stroke | No meta-analysis |
| 158 | Yang, Seoyon and Suh, Jee Hyun and Kwon, SuYeon and Chang, Min Cheol | The effect of neurologic music therapy in patients with cerebral palsy: A systematic narrative review | No meta-analysis |
| 159 | Yoo, P. and Mogo, E. and Bergthorson, M. and McCabe, J. and Shikako-Thomas, K. and Majnemer, A. | The impact of context-based interventions on participation of children with disabilities: A systematic review | No meta-analysis |
