# Supplementary material for: Strategies for addressing the needs of children with or at risk of developmental disabilities in early childhood by 2030: a systematic umbrella review
Source: BMC Med. 2024 Feb 2;22:51. doi: 10.1186/s12916-024-03265-7 (PMC10835858; doi:10.1186/s12916-024-03265-7)
Supplement: Supplementary file 3 — Additional file 3. Study characteristics of reviews with nonaggregate data, for children of any age. [file 12916_2024_3265_MOESM3_ESM.docx]

**Additional file 3: Study characteristics for children of any age**

| First author (year) (ref) | Target disability | Evidence based intervention | Intervention details | Age range | Country - n | Outcomes and impact | Participants | Overall confidence |  |
| --- | --- | --- | --- | --- | --- | --- | --- | --- | --- |
| **Motor impairment** | | | | | | | | | |
| Blumetti (2019) (40) | CP | Early intervention and rehab | BoNT-A injections in the lower limb muscles compared to other interventions | birth to 19 years | USA – 5, Australia - 6 Netherlands - 2 Chile – 1, Sweden - 1 Egypt – 1, India - 1 Turkey – 2, Jordan - 1 Ireland – 1, Poland - 1 UK – 2, China - 2 Multiple - 5 | BoNT-A is not more effective than ankle serial casting to treat ankle contractures, but is more effective than orthotics at improving range of motion and spasticity. | 31 studies, 1,508 participants | High |  |
| Miyahara (2017) (41) | Developmental co-ordination disorder | Early Intervention | Task oriented intervention | 4 - 18 years | Australia - 2 Taiwan - 2  Sweden - 1 UK - 1 | Task oriented interventions improve motor co-ordination compared to no intervention: MD -3.63 (95%CI -5.88 - -1.39) | 6 studies, 169 participants | High |  |
| Smits Engelsman (2012) (42) | Developmental co-ordination disorder | Early intervention | Early intervention to improve motor performance | All ages | Unclear | Mean ES increased across all treatments: 0.56, 95%CI: not in supplementary tables | 26 studies, 912 participants | Low |  |
| Yu (2018) (43) | Developmental co-ordination disorder | Early intervention | Motor skill interventions | 3 - 17 years | Hong Kong - 5 Singapore – 1, Australia - 2 Ireland - 1 UK – 2, Canada - 2 South Africa – 1, Sweden – 1, Taiwan - 3 | Motor performance improved 0.63 (95%CI: 0.31 - 0.94) | 18 studies, Unclear | Low |  |
| **Cognitive Impairment** | | | | | | | | | |
| Ahn (2018) (44) | Neurodevelopmental disorders | Early intervention and rehab | Cognitive rehabilitation of adaptive behaviour | 0-14 years | USA – 4, Taiwan – 1, Netherlands - 1 | Cognitive therapy improves adaptive behaviour: 0.64 (95%CI: 0.40 - 0.87) | 6 studies, 549 participants | Moderate |  |
| May (2021) (45) | ID and ASD | Early intervention | Dance | 3 - 18 years | USA – 9, Poland - 2 Turkey - 2 Brazil – 1, Greece - 1 Japan - 1 South Korea - 1 Taiwan – 1, UK - 1 | Improved balance: SMD = 1.03, 95%CI: 0.58 - 1.48) and jumping: SMD = 0.71, 95%CI: 0.26 - 1.16 | 19 studies, 521 children | Moderate |  |
| McGarty (2018) (46) | ID | Early intervention | Interventions to increase physical activity | 0-18 years | Australia - 1 USA - 3 New Zealand - 1 | Intervention groups not more effective at increasing physical activity (d: 2.20; 95% CI: 0.57 - 0.97), due to a decrease in PA in the control intervention, effect was demonstrated at follow-up (d: 0.49; 95% CI 0.14 - 0.84). | 5 studies , 191 participants | Low |  |
| Reichow (2019) (47) | ID | Early Intervention | Reading interventions | 4 - 12 years | USA - 4 UK - 2 Canada - 1 | Phonologic awareness SMD 0.55, 95% CI 0.23 - 0.86; 4 studies, 178 participants. word reading SMD 0.54, 95% CI 0.05 - 1.03; 5 studies, 220 participants. oral reading fluency SMD 0.65, 95% CI –0.12 - 1.42; 2 studies, 84 participants. language skills (SMD 0.28, 95% CI 0.03 to 0.54; 3 studies, 222 participants | 7 studies, 352 children and adolescents | High |  |
| **Behavioural** | | | | | | | | | |
| Brignell (2022) (48) | ASD | Early intervention | Pharmaceutical - Memantine, aim to reduce core symptoms | All ages | USA - 2 Iran - 1 | No clear evidence for a difference in reduction in core symptoms when comparing memantine to placebo (standardised mean difference) SMD -0.74 (95% CI -2.07 - 0.58) | 2 studies, 181 participants | High |  |
| Eckes (2023) (49) | ASD | Early intervention | Applied Behavioural analysis | All ages | Norway - 3  Italy - 1 Sweden - 1 USA -2, UK -2 Germany -1, Israel - 1 | Applied behaviour analysis has medium effects compared to treatment as usual/minimal/no treatment for intellectual functioning SMD = 0.51 (95% CI 0.09 -0.92) and adaptive behaviour SMD = 0.37 (95% CI 0.03 - 0.70). | 11 studies, 632 participants | Moderate |  |
| Fuller (2020a) (50) | ASD | Early intervention | Early intervention for social communication outcomes | All ages | USA - 24 UK- 4 Australia - 1 | Children in early interventions showed greater improvements on measures of social communication compared to controls ES = 0.36 (95% CI 0.21–0.50) | 29 studies, 1,442 children | Moderate |  |
| Groenman (2021) (51) | ADHD | Early intervention | Behavioural interventions that impact symptoms, oppositional defiant disorder and conduct disorder | All ages | Iran - 1 USA - 16 UK - 3 Israel - 1 Netherlands - 2 New Zealand -2* | Behavioural interventions reduce ADHD symptoms: Effect size -.42 (no CI)(parenting and child interventions), inattention symptom severity decreased = ES -.46 (no CI) and hyperactivity = ES -.27 (no CI) | 21 studies, 2,233 participants | High |  |
| Liu (2020) (52) | ASD | Early intervention | Parent mediated intervention | 1 – 15 years | China, Hong Kong, Taiwan | Parent mediated interventions improved language communication: SMD = 0.64 (95% CI: 0.48–0.81), social competence: SMD = 0.63 (95% CI: 0.44–0.83), adaptive behaviour: SMD = 0.68, (95% CI: 0.11–0.81) compared to controls | 12 studies, 964 children | High |  |
| Naveed (2019) (53) | ASD | Early intervention | Non-specialist mediated interventions | 0 - 17 years | Australia - 3 USA - 20 India - 1 Belgium -1 UK - 2 Norway - 1 Canada - 2 Netherlands - 1 Multiple - 1 | Non specialist mediated interventions improve function overall: 0.44 (95%CI: 0.27 - 0.60) and child distress: SMD = 0.55 (95% CI = 0.25 - 0.85), communication: SMD = 0.23 (95% CI = 0.03 to 0.42), expressive language: SMD = 0.47 (95% CI = 0.22 to 0.72), motor skills: SMD = 0.25 (95% CI = 0.02 to 0.48), repetitive behaviors: SMD = 0.33 (95% CI = 0.05 to 0.62), social skills: SMD = 0.53 (95% CI = 0.34 to 0.73), symptom severity: SMD = 0.44 (95% CI = 0.27 to 0.60) and visual reception: SMD = 0.29 (95% CI = 0.01 to 0.57) | 32 studies, total not reported | High |  |
| Randall (2018) (54) | ASD | Early detection | Diagnostic test accuracy | 12 months - 8 years | USA - 14 UK - 2 Australia - 2 Netherlands - 2 India - 1 | Autism Diagnostic Observation Schedule - Generic summary sensitivity was 0.94 (95% CI 0.89 to 0.97), summary specificity was 0.80 (95% CI 0.68 to 0.88). Childhood Autism  Rating Scale summary sensitivity for CARS was 0.80 (95% CI 0.61 to 0.91), and summary specificity was 0.88 (95% CI 0.64 to 0.96). summary sensitivity for Autism Diagnostic Interview - Revised was 0.52 (95% CI 0.32 to 0.71), and the summary specificity was 0.84 (95% CI 0.61 to 0.95). | ADOS: 12 studies, 1625 children. CARS: 4 studies, 641 children. ADI-R: 5 studies, 634 children. | High |  |
| Sandbank (2022) (55) | ASD | Early Intervention | Behavioral, developmental, NDBI, TEACCH, sensory-based, animal-assisted, and technology-based interventions | under 8 years | Unclear | Improved social communication: 0.31 (95%CI: 0.09 - 0.54) Play: 0.33 (0.13 - 0.53) | Unclear | Low |  |
| Shen (2023) (56) | acquired brain injury | Early Intervention | Family-oriented interventions | 0 - 8 years | USA – 25, Brazil - 2 Australia - 3 Mexico – 1, Israel - 1 Multiple - 2 Canada - 2 | Improved child outcome (14 studies) g = 0.43 (95% CI: 0.11- 0.76) improved parental outcomes g = 0.45 (95% CI:0.16 - 0.74) | Child outcomes: 14 studies, 1145 participants parental outcomes: 11 studies, 931 participants | Moderate |  |
| Shi (2021) (57) | ASD | Early Intervention | Early comprehensive models: intensive behavioral intervention (EIBI), and  Early Start Denver Model (ESDM) | 5 - 18 years | USA - 12 UK - 3 Australia - 2 Canada - 1 | ESDM IQ improvement effect size (ES) = 1.37 (95% CI: 0.95 to 1.80), EIBI symptom reduction ES −1.27, (95% CI: −1.96 to −0.58) | 18 studies, 495 children | High |  |
| Storebo (2023) (58) | ADHD | Early intervention | Methylphenidate | 3 to 18 years | Germany - 1 Israel - 1 USA – 9, Canada - 3 Multiple - 3 Netherlands - 2 UK – 2, Not stated - 1 | Improved teacher-rated ADHD symptoms SMD: - 0.74, (95% CI: −0.88 to −0.61) | 21 studies; 1728 participants | High |  |
| Uljarevic (2022) (59) | ASD | Early intervention | Pivotal response treatment | all ages | Unclear | Improved language and communication outcomes: Expressive (3 studies; SMD:0.31, 95% CI: 0.04 - 0.58), Receptive (3 studies; SMD:0.51, 95% CI: 0.23 - 0.80) | 8 studies, Unclear | Low |  |
| Wang (2023) (60) | ADHD | Early intervention | Physical activity | < 18 years | USA – 1, Iran - 2  Switzerland - 2 Multiple - 2 Tunisia – 3, Korea - 1 | Inhibitory function of children with ADHD improved (SMD): 0.78, 95% CI: 0.45–1.10) | 11 studies, 713 participants | Moderate |  |
| Wergeland (2022) (61) | ASD | Early intervention | Early behavioural interventions | under 8 years | USA – 11, Israel - 4 UK – 5, Sweden - 2 Norway - 3 Greece - 1 Romania - 1 Australia - 2 | Skills improved in: Adaptive 0.81 [95%CI: 0.50 - 1.12]  Cognitive 0.76 [95%CI: 0.57 - 0.96]  Communication 1.27 [95%CI:1.00 - 1.54]  Socialization 1.01 [95%CI: 0.76 - 1.26] | 29 studies, 1422 participants | High |  |
